# Self‐Orienting Hydrogel Micro‐Buckets as Novel Cell Carriers

**DOI:** 10.1002/anie.201811374

**Published:** 2018-11-27

**Authors:** Qian Liu, Meng Zhao, Serhii Mytnyk, Benjamin Klemm, Kai Zhang, Yiming Wang, Dadong Yan, Eduardo Mendes, Jan H. van Esch

**Affiliations:** ^1^ Department of Physics Beijing Normal University Beijing 100875 P. R. China; ^2^ Department of Chemical Engineering Delft University of Technology van der Maasweg 9 Delft 2629 HZ The Netherlands; ^3^ Department of Materials Science and Engineering Delft University of Technology Mekelweg 2 Delft 2628 CD The Netherlands

**Keywords:** cell carriers, hydrogels, microfluidics, self-orientation, soft matter

## Abstract

Hydrogel microparticles are important in materials engineering, but their applications remain limited owing to the difficulties associated with their manipulation. Herein, we report the self‐orientation of crescent‐shaped hydrogel microparticles and elucidate its mechanism. Additionally, the microparticles were used, for the first time, as micro‐buckets to carry living cells. In aqueous solution, the microparticles spontaneously rotated to a preferred orientation with the cavity facing up. We developed a geometric model that explains the self‐orienting behavior of crescent‐shaped particles by minimizing the potential energy of this specific morphology. Finally, we selectively modified the particles’ cavities with RGD peptide and exploited their preferred orientation to load them with living cells. Cells could adhere, proliferate, and be transported and released in vitro. These micro‐buckets hold a great potential for applications in smart materials, cell therapy, and biological engineering.

Hydrogel microparticles are widely used in materials science and biological engineering with applications ranging from encapsulation and delivery of cells and pharmaceuticals to catalysis and scavenging of molecules.^1^ Biocompatible hydrogel microparticles have been previously produced using such methods as electrohydrodynamic jetting,^2^ micromolding,^3^ and microfluidics.^4^ Among them, microfluidics is one of the most promising methods as it allows generation of multi‐compartmentalized microparticles with precise control of dimensions and morphologies,^5^ such as Janus‐type,^6^ core–shell,^7^ and so‐called “crescent‐shaped” particles, that possess open cavities.^8^ There have been attempts to use the crescent‐shaped microgels for confining enzymatic reactions for particle propulsion,^9^ or for selective capture microscale objects, such as smaller particles or algae cells.^10^ However, the efficient loading of cargo like particles or living cells into the cavity has proven to be complex owing to the lack of suitable long range interactions that extend beyond the cavity size, and the difficulties associated with simultaneously manipulating the cargo and target cavity opening. Therefore, new approaches that enable the efficient loading of cargo into cavities of microparticles like crescent‐shaped particles are of great interest for their application as, for example, microcarriers and functional micro‐objects in engineering and biology.

Herein, we show that self‐orienting crescent‐shaped hydrogel particles can be used as micro‐buckets, which can easily be loaded with cargo such as living cells and used to transport and release their cargo. We found that owing to their morphology, crescent micrometer‐sized particles spontaneously rotate under the action of gravity to exhibit a preferred orientation with the cavity facing up. By exploiting this feature, we have successfully loaded these micro‐buckets with cells, and then cultured and manipulated the cells in the micro‐buckets. We expect our findings to find use in the fields of cell therapy and in design of smart biomaterials.

As shown in Scheme 1 a, the crescent‐shaped particles were produced by using aqueous two‐phase systems (ATPS) in a microfluidic device followed by selective photo‐cross‐linking of one component (Supporting Information, Figure S1 and Video 1).8a When the crescent‐shaped gel particles were pipetted into a petri dish and allowed to settle, the particles spontaneously rotated from a random distribution to a preferred orientation under the influence of gravity. This self‐orienting behavior allows easily controlling these microparticles and facilitates the efficient loading of materials. We functionalized the inner surface of the microparticles with cell‐adhesive RGD peptide and demonstrated these self‐orienting gel particles could carry living cells as micro‐buckets. As shown in Scheme 1 b, owing to the preferred orientation of cavity facing up, cell loading can be easily and efficiently accomplished. After cell adhesion and/or proliferation, the cells in the hydrogel micro‐buckets can be transported and released.

**Scheme 1:**
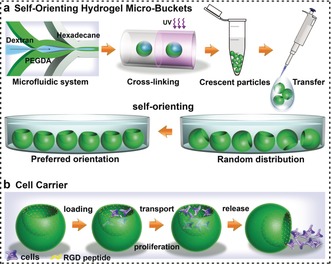
a) Schematic of the fabrication of crescent‐shaped hydrogel microparticles using microfluidics and their self‐orientation under water. b) Peptide‐modified hydrogel micro‐bucket as cell carriers for cell loading, transport, proliferation, and release.

The morphology of crescent‐shaped hydrogel microparticles was observed by scanning electron microscopy (SEM) and confocal laser scanning microscopy (CLSM), as illustrated in Figure 1 a. The micrographs show that the dextran phase was completely removed, leaving the hydrogel microparticle featuring an open cavity. The size of the crescent‐shaped microparticles could be measured by the bright‐field microscopy. As shown in Figure 1 b, the particle diameter (black) is 2*R*, cavity diameter (green) is 2*r* and the opening size (white) is *d*_l_. A variety of crescent‐shaped hydrogel microparticles with different cavity sizes were obtained by varying the volumetric flow rate ratios of the dextran and PEGDA phases (*F*_d_:*F*_p_). As shown in Figure 1 c, 2*R* was in the range of 125–142 μm. With the decreasing *F*_d_:*F*_p_, 2*r* and *d*_l_ decreased, and smaller cavities and thicker shells were obtained.

**Figure 1:**
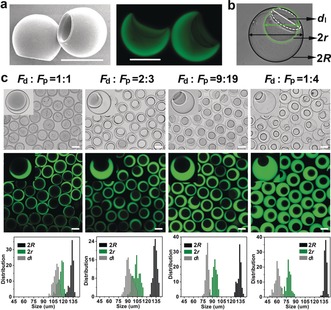
a) Crescent‐shaped hydrogel microparticles imaged by SEM after freeze‐drying (left) and CLSM (right). b) Bright‐field microscopy image showing the particle diameter (2*R*), cavity diameter (2*r*), and opening size (*d*_l_) of a crescent‐shaped hydrogel microparticle. c) Crescent‐shaped hydrogel microparticles with four different cavity sizes imaged by using bright‐field microscopy (top) and CLSM (middle) prepared by different volumetric flow rate ratios of dextran and PEGDA (*F*_d_:*F*_p_), and the corresponding size distributions (bottom). PEGDA phases were labelled with fluorescein methacrylate (green). Scale bars=100 μm.

Interestingly, inspection of microscopy images of the sedimentary crescent‐shaped hydrogel microparticles revealed that most microparticles exhibited a preferred orientation with the cavity opening facing upwards (see Figure 1 c). To better understand the spontaneous orientation, we tracked the microparticles sedimentation (see Video 2). When the hydrogel particles reached the bottom (before achieving equilibrium), they had no obvious preferred orientation. However, at the bottom, the particles spontaneously rotated until the cavity pointed upwards (after achieving equilibrium), resulting in the preferred orientation. We can therefore hypothesize that the driving force of this rotation is the gravitational force, while the friction with the solvent and neighboring particles does not prevent the particles from adopting a preferred orientation.

To verify this hypothesis and investigate possible gravitational effects on the orientation of micrometer‐sized crescent‐shaped particles, a geometric description of crescent‐shaped particle was developed. As shown in Figure 2 a, we applied a two‐dimensional coordinate system in the mirror plane of the particles (best viewed from the particle side) and assumed that the crescent‐shaped gel microparticles were formed by two spheres with centers C_1_ and C_2_, and radii *R* and *r* (large and small spheres, respectively). The opening of the cavity is a disk with diameter *d*_l_, and the rotation angle of the particle relative to the direction of the gravitational force is given by *θ*. When viewed from the top, the cavity opening appears as an ellipse (see Figure S2) with the ratio between the short (*d*_s_) and long axis (*d*_l_) depending on the rotation angle of the particle. Hence, the rotation angle can be calculated as cos(*θ)*=*d*_s_/*d*_l_. When the cavity opening of the particle points upwards, *θ*=0°, *d*_s_=*d*_l_, and cos(*θ)*=1, and when the cavity opening of the particle points side‐wards, *θ*=90°, *d*_s_=0, cos(*θ)*=0. It should be noted that when the particle rotates more than 90°, the rotation angle is 180°−*θ*. In the calculation, *R*, *r*, and *d*_l_ come from the size distributions (Figure 1 c) and are the average values for a set of particles, with *R* normalized to 100 μm. From this geometrical model, the heights of the center of mass for various rotation angles are shown in the Figure S4. Potential energy changes (Δ*E*_p_) for various rotation angles were calculated from the equations (see Supporting Information, Equations 1, 2, and 3) and illustrated in Figure 2 b. Obviously, at *θ*=0°, the particle had the lowest potential energy, which gradually increased with *θ*, until *θ* reached a value of circa 140° to 150°, its precise value depending on the particle size. Above this value of *θ*, the potential energy decreased again and reached a local minimum at *θ*=180°, which is the upside‐down state. This represents a metastable state in which particles can remain kinetically trapped. Nevertheless, particles trapped in the upside‐down state were rarely observed, most likely because during the sedimentation, the particles already pre‐orient their center of mass towards the lowest energy state.

**Figure 2:**
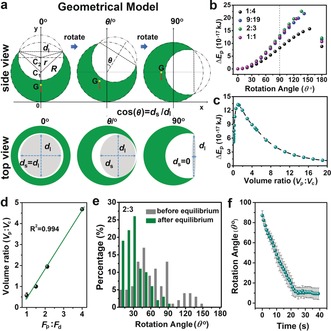
a) Geometric model of the crescent‐shaped hydrogel microparticle. Potential energy change of various cavity‐sized particles at different b) rotation angle and c) volume ratio (*V*_p_:*V*_c_). d) The relationship between volumetric flow rate ratio (*F*_p_:*F*_d_) and volume ratio (*V*_p_:*V*_c_). e) Rotation angle distributions of particles (*F*_d_:*F*_p_=2:3) before (grey) and after (green) achieving equilibrium. f) Time scale of particle rotation, the results are shown as the mean±s.d. of 10 microparticles. The statistics are based on 100 crescent‐shaped hydrogel microparticles.

At the same time, based on the model, we also calculated Δ*E*_p_ (from *θ*=0° to *θ*=90°) for the particles with different cavities. As shown in Figure 2 c, the particle radius was 100 μm and cavity size can be represented by their volume ratio (volume of particle divided by volume of cavity, *V*_p_:*V*_c_). Δ*E*_p_ first increased to a maximum value and then decreased with the increase of volume ratio. This indicates that the particles with a volume ratio in the range of 1–2.5 have the largest energy gain for the cavity facing up. The model shows that, as to be expected, Δ*E*_p_ increases with *R* and density (Figure S5). Meanwhile it also predicts that the particles may lose the preferred orientation when *R* decreases to values smaller than circa 5 μm when Δ*E*_p_ becomes comparable to *kT* at 298 K.

As a first validation of the model, we compared the particle and cavity volume ratio (*V*_p_:*V*_c_) with volumetric flow rate ratios (*F*_p_:*F*_d_). The *V*_p_:*V*_c_ was calculated by the geometrical description of the particles given by Equation 1 (Supporting Information) with the experimentally observed size distributions for particles produced with different *F*_p_:*F*_d_. We observed a linear relationship between *V*_p_:*V*_c_ and *F*_p_:*F*_d_ (Figure 2 d). The excellent correlation indicates that the model accurately describes the crescent‐shaped particle geometry in terms of *R*, *r* and *d*_l_. Moreover, particle geometry can be accurately tuned by adjusting the ratio of volumetric flow rates.

Next, we used the model to obtain the rotation angle distributions of the crescent‐shaped particles before and after reaching their equilibrium orientations. The rotational angles of individual particles were calculated from the experimental ratios between *d*_s_ and *d*_l_. As shown in Figure 2 e, when the particles had just settled at the bottom, *θ* was randomly distributed over a wide angular range from 0° to 150°. However, immediately after settling, the particles started to rotate and within approximately 30 s (Figure 2 f) they came to rest in a new orientation, now with about 70 % of the particles having rotation angles in the range of 0° to 30° and over 90 % having rotation angles in the range of 0° to 60°. Similar angular distributions were obtained for the crescent‐shaped particles with other cavity sizes (Figure S6). From the angular rotation velocity (around 4° s^−1^, Figure 2 f), the frictional force was estimated to be 1.2×10^−11^ N (see Figure S7), which is much smaller than the gravitational force (4.2×10^−8^ N). These results clearly indicate that gravity is the main driving force leading to the upward orientation of the crescent‐shaped particles, while possible anisotropic frictional forces, which are expected to be present during sedimentation or rotation, apparently play a minor role in this process.

Based on the preferred upward‐facing orientation of the cavity, we anticipated that these self‐orienting crescent‐shaped hydrogel microparticles could be used as micro‐buckets for cell loading and transport after specific functionalization. As shown in Figure 3 a, the RGD peptide, a widely‐used cell‐adhesive peptide,^11^ was added to the dextran phase. Based on the partition coefficient of RGD peptide in ATPS (*K*_p_=2.4, Figure S8), the peptide preferred the PEGDA phase, leading to the RGD peptide being mostly immobilized in the cavity of the hydrogel microparticles through a thiol‐ene click reaction.^12^ In order to load cells into the gel buckets, a suspension of mouse fibroblast cells (NIH/3T3) was added to the cell‐culture plate containing a layer of oriented functionalized hydrogel micro‐buckets. After cell deposition, cells were present in the cavities as well as on the glass bottom of the culture plate. The fraction of particles loaded with cells and the number of cells in the particles could be adjusted by changing the density of suspended cells, and under optimized conditions, more than 98 % particles could be loaded with 6–10 cells per particle (see Figure S9). Cells that have entered the cavities readily adhered to the interior and spread along the walls as observed from cell‐shape change (Figure 3 b), while the cells outside the buckets adhered to and spread on the glass (Figure S10). To demonstrate the presence and function of RGD peptide on the inner surface of the micro‐buckets, we carried out control experiments with identical unmodified microparticles. In this case, cells retained a spherical shape and did not spread in the cavity (Figure 3 c) owing to well‐known anti‐biofouling properties of PEGylated surfaces.^13^

**Figure 3:**
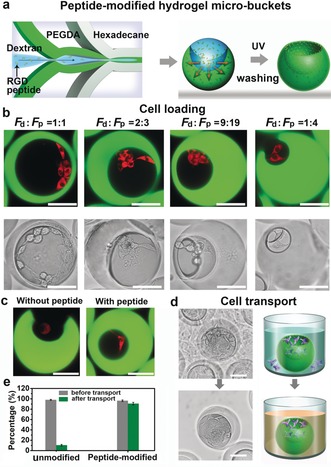
a) Schematic of the fabrication of peptide‐modified hydrogel micro‐buckets. b) CLSM (top) and bright‐field (down) microscopy images of the different cavity‐sized micro‐buckets after cell loading. c) CLSM microscopy images of cells in the hydrogel micro‐bucket (*F*_d_:*F*_p_=9:19) with (right) and without peptide (left). d) Bright‐field microscopy images and schematic of cell transport. e) The ratio of cell adhesion in unmodified and peptide‐modified microparticles before (grey) and after transport (dark green). The results are shown as the mean±s.d. of three independent experiments, 200 particles (*F*_d_:*F*_p_=1:1) for each experiment. The cells were labelled with CellTracker red CMTPX. Scale bar=50 μm.

We expected that these micro‐buckets loaded with adhered cells would allow us to easily transfer them without the need of detaching/dissociating the cells using proteases. Indeed, the cells in the cavity could be easily isolated for further investigation by simple pipetting of the cell‐laden micro‐buckets into a new culture plate, while the cells outside remained in the previous culture plate (Figure 3 d). After transport, only about 10 % unmodified microparticles had cells, while about 90 % peptide‐modified microparticles retained the cells in the cavity interior (see Figure 3 e). Moreover, no cell attachment to the outer surface of the particles was observed, suggesting that the cavity exterior surface was not sufficiently decorated with RGD peptide. These results confirm the successful immobilization of RGD peptide on the inner surface of the hydrogel micro‐buckets and its role in cell adhesion and also demonstrate that these peptide‐modified hydrogel micro‐buckets can retain cells during transport.

In addition to cell loading and transport in these functionalized hydrogel micro‐buckets, we could also achieve cell proliferation and release. As shown in Figure 4 a, a peptide‐modified hydrogel micro‐bucket with cells was tracked for several days. After 3 days, cells adhered to the hydrogel and spread in the cavity. In the period between 5 to 14 days after seeding, the number of cells in the cavity increased, while they remained attached to the inner surface of the cavity and were connected to the neighboring cells. As shown in Figure 4 b, cell viability in the control experiments was around 98 % at 4, 8, and 12 days. In the gel buckets cell viability slightly decreased to 94 % after 12 days of cell culture. This indicates that the cells remain alive in the peptide‐modified hydrogel micro‐buckets.

**Figure 4:**
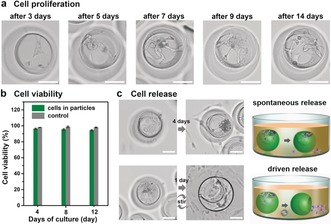
a) Bright‐field microscopy images of cells in one particle after different days of cell culture. b) Cell viability in hydrogel microparticles (dark green) and cell culture plate (grey) after different days. Viability of cells was quantified as the percentage of the alive cells. The results are shown as the mean±s.d. of three independent experiments, 100 particles (*F*_d_:*F*_p_=2:3) for each experiment. c) Bright‐field microscopy images and schematic of cell release. Scale bar=50 μm.

After cell proliferation, the cells could also be released from the hydrogel micro‐buckets in several ways (Figure 4 c). Most commonly, spontaneous release of cells occurred when the cavity became nearly filled with cells as a consequence of proliferation (see Figure S14). After 4 days, a few cells escaped from the hydrogel microparticle, while most of the cells stayed in the cavity. We speculate that the cells detach from the particles because the outer surface of the hydrogel particles has insufficient RGD peptide for cell adhesion. Additionally, we could release cells from the micro‐buckets by continuous gentle agitation of the particle–cell suspensions, leading to a fast and complete release of the cells. After being released, the cells were still alive with the cell viability around 93 % (Figure S15).

In conclusion, we developed the self‐orienting crescent‐shaped hydrogel microparticles, explained the mechanism of their self‐orientation, and used them as micro‐buckets to carry living cells in vitro. These crescent‐shaped particles were produced by microfluidic generation of ATPS droplets, followed by selective cross‐linking of PEGDA phase and removal of the dextran phase. The cavity size of hydrogel microparticles could be regulated by varying the flow rate of each solution. Because of the specific morphology, these crescent‐shaped hydrogel microparticles spontaneously oriented with the cavity facing up under the influence of gravity. To utilize this preferred orientation, we demonstrated a potential application of these particles as cell carriers. By modifying the particles’ cavities with cell‐adhesive peptide, we successfully demonstrated in vitro cell loading, transport, proliferation and release, indicating that these self‐orienting hydrogel micro‐buckets hold great promise for applications in many bio‐engineering processing, cell delivery, and smart biomaterials as well as providing a new tool in fundamental biological studies.

## Conflict of interest

The authors declare no conflict of interest.

## Supporting information

As a service to our authors and readers, this journal provides supporting information supplied by the authors. Such materials are peer reviewed and may be re‐organized for online delivery, but are not copy‐edited or typeset. Technical support issues arising from supporting information (other than missing files) should be addressed to the authors.

Supplementary

Supplementary

Supplementary
