# Supplementary material for: Self‐Orienting Hydrogel Micro‐Buckets as Novel Cell Carriers
Source: Angew Chem Int Ed Engl. 2018 Nov 27;58(2):547–51. doi: 10.1002/anie.201811374 (PMC6391985; doi:10.1002/anie.201811374)
Supplement: Supplementary file 1 — Supplementary [file ANIE-58-547-s001.pdf]

Angewandte Chemie
Eine Zeitschrift der Gesellschaft Deutscher Chemiker

Supporting Information

Self-Orienting Hydrogel Micro-Buckets as Novel Cell Carriers

*Qian Liu, Meng Zhao, Serhii Mytnyk, Benjamin Klemm, Kai Zhang, Yiming Wang, Dadong Yan, Eduardo Mendes, and Jan H. van Esch**

anie_201811374_sm_miscellaneous_information.pdf
anie_201811374_sm_Video_1.mp4
anie_201811374_sm_Video_2.mp4

Content

Video 1: Formation of the crescent-shaped hydrogel microparticles

Video 2: Self-orientation of the crescent-shaped hydrogel microparticles

1. Materials

2. Characterization

3. Fabrication of microfluidic device

4. Formation of the crescent-shaped hydrogel microparticles

5. Measuring of d_s and d_l

6. Modelling

7. Partition coefficient of RGD peptide in ATPS

8. Bio experiment

Cell staining

Cell culture

Cell viability

Cells in different sized hydrogel microparticles

Cell proliferation and release

Cell viability after being released

1. Materials

Poly(ethylene glycol) diacrylate (PEGDA, M_W=700), dextran (M_W=20000), sodium hydroxide, span 80, 4-(2-hydroxyethyl)piperazine-1-ethanesulfonic acid (HEPES), fluorescein methacrylate and hexadecane were purchased from Sigma-Aldrich (Steinheim, Germany). Cyclo(Arg-Gly-Asp-D-Phe-Cys) (RGD peptide) was purchased from Peptides International, Inc (Kentucky, USA). CellTracker™ red CMTPX, Hoechst 33342 and propidium iodide (PI) were purchased from Thermo Fisher Scientific Inc. (Landsmeer, Netherlands). The initiator lithium phenyl-2,4,6-trimethylbenzoylphosphinate (LAP) was synthesized as described previously.[1] NIH/3T3 cell (mouse fibroblast cells) culture line was obtained from American Type Culture Collection.

2. Characterization

Microfluidic experiments were performed on Axio Observer A1 inverted microscope (Zeiss, ×10 air objective) with a Zyla 5.5 sCMOS camera (Andor) at 50 fps. Size distributions, self-orientation and cell loading were obtained by confocal laser scanning microscopy (CLSM, Zeiss LSM 710, ×10 and ×20 air objectives and a ×40 oil immersion objective). Mercury-arc light source (HXP 120 V,120 W) with a band pass filter 300-400 nm (peak intensity at 365 nm) was used to supply UV light. Particles morphology was checked by JEOL 6010 Scanning Electron Microscope (SEM) after freeze-drying in liquid nitrogen. Cell transport, proliferation, release and viability were obtained by Andor Inverted Microscope (Zeiss, ×20 air objective).

3. Fabrication of microfluidic device

The device was fabricated by PDMS (Dow Corning, Sylgard 184 elastomer kit) using soft lithography. The non-planar chip was bonded by two pieces of PDMS after oxygen plasma treatment. The channel height is about 300 μm, width is about 500 μm. The width of nozzles is about 40 μm. The device was connected to individual syringe pumps (Harvard Apparatus, 11PicoPlus) via tube (PEEK® 0.5/1.6 mm inner/outer diameter).

4. Formation of the crescent-shaped hydrogel microparticles

All solutions were prepared using demineralized water. Dextran (28.6% W/W) and RGD peptide (4 mg/mL) were dissolved in HEPES buffer (50 mM, pH=7) solution. PEGDA (28.6% W/W) and LAP (10 mg/mL) were dissolved in demineralized water. Surfactant (span 80, 3% w/w) was dissolved in hexadecane. These three phases were independently injected into the microfluidic device by syringe pumps. As shown in **Figure S1a and S1b**, Dextran and polyethylene glycol diacrylate (PEGDA) with photo initiator were used as inner phase and middle phase, respectively. Co-flows of dextran and PEGDA break up into highly monodisperse phase separated ATPS droplets in hexadecane flow. The volumetric flow rate of dextran phase was 0.03~0.1 μL/min and that of PEGDA phase was 0.1~0.12 μL/min. The volumetric flow rate of hexadecane was 12~15 μL/min.

Fluorescein methacrylate was added into PEGDA phase, and the fluorescence images (**Figure S1c and S1d**) indicate that PEGDA forms the outside (the crescent part), and dextran forms the inside of the particles (the spherical part). The hydrogel particles were cross-linked by on chip UV irradiation and collected into vials. Firstly, hexadecane in the vial was removed by pipetting. Secondly, the particles were washed with THF (3 times) and water (5 times) to remove surfactant and dextran. Lastly, the hydrogel particles were washed with ethanol (3 times). Then the hydrogel particles were prepared for the bio experiment after ethanol evaporation.

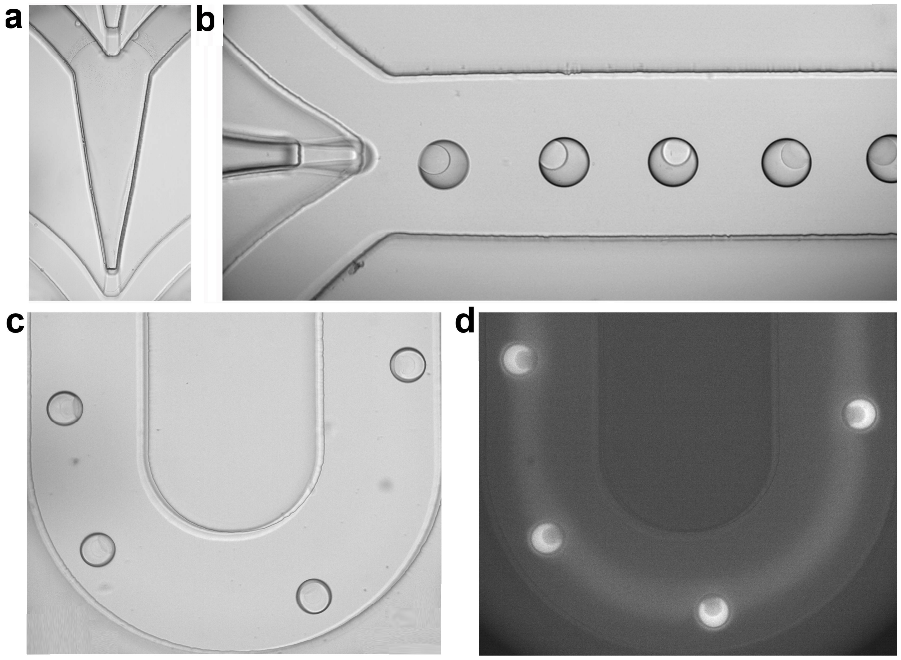

Figure S1. Formation of crescent-shaped hydrogel microparticles. (a) Microscopy images of the injection nozzles structure. **(b)** Generation of ATPS droplets. Microscopy images of phase-separated hydrogel particles with **(c)** and without **(d)** visible light during UV cross-linking. Fluorescein methacrylate was added into PEGDA phase.

5. Measuring of d_s and d_l

From the bright-field microscopy image of the crescent-shaped hydrogel microparticle, the rotation of particle causes an ellipse (white dash, **Figure S2**). Since, the short diameter (d_s, red line) and the long diameter (d_l, white line) of the ellipse can be measured by the software.

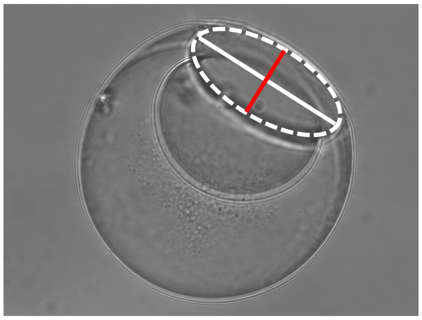

Figure S2. Measuring of d_s (red line) and d_l (white line) on the bright-field microscopy image.

6. Modelling

We established a geometrical model (as shown in **Figure S3a)** to calculate the center of mass and potential energy of the crescent-shaped hydrogel microparticle at different rotation angle. We assume that the crescent-shaped hydrogel microparticles are formed by two spheres with two centers (2 and 3) and radiuses (*R* and *r*). A two-dimensional coordinate system was established based on the side view of the particle. The X axis is the tangent line of the big sphere of the particle, while the Y axis is along the connection between the centers of the two spheres. Therefore, the bottom point of the particle is placed at the origin of the coordinate system. Meanwhile, the rotation angle of particle is θ. From the top view, the rotation angle can be calculated as $\cos(\theta)=d_s/d_l$ (short diameter divided by long diameter). The specific coordinates of Y_0-Y_6 in the side view are given in **Figure S3b**.

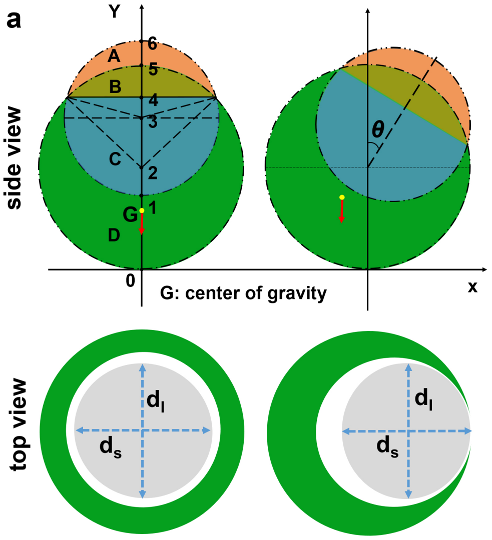

b

$$Y_0 = 0; \quad Y_2 = R; \quad Y_5 = 2R; \quad \cos\theta = d_s / d_l;$$

$$Y_1 = R + \sqrt{R^2 - \left(\frac{d_l}{2}\right)^2} - \sqrt{r^2 - \left(\frac{d_l}{2}\right)^2} - r$$

$$Y_3 = R + \sqrt{R^2 - \left(\frac{d_l}{2}\right)^2} - \sqrt{r^2 - \left(\frac{d_l}{2}\right)^2}$$

$$Y_4 = R + \sqrt{R^2 - \left(\frac{d_l}{2}\right)^2}$$

$$Y_6 = R + \sqrt{R^2 - \left(\frac{d_l}{2}\right)^2} - \sqrt{r^2 - \left(\frac{d_l}{2}\right)^2} + r$$

Figure S3. Modelling for the crescent-shaped hydrogel microparticle. (a) Geometrical model of the crescent-shaped hydrogel microparticle. **(b)** Relationship between particle radius (*R*), cavity radius (*r*), opening size (d_l) and rotation angle (θ).

Volume of the crescent-shaped hydrogel microparticle (Equation 1):

$$V = \int S \, \mathrm{d}Y$$

$$\begin{cases} S = \pi\left[R^2 - (Y - Y_2)^2\right] - \pi\left[r^2 - (Y - Y_3)^2\right] & (Y1 \le Y \le Y4) \\ S = \pi\left[R^2 - (Y - Y_2)^2\right] & (Y_0 \le Y \le Y_1) \end{cases}$$

The mass center of the particle (G, θ=0º) can be estimated by the integration along the Y axis:

Height of mass center (Equation 2):

$$G = \frac{\int Y \cdot S \, \mathrm{d}Y}{\int S \, \mathrm{d}Y}$$

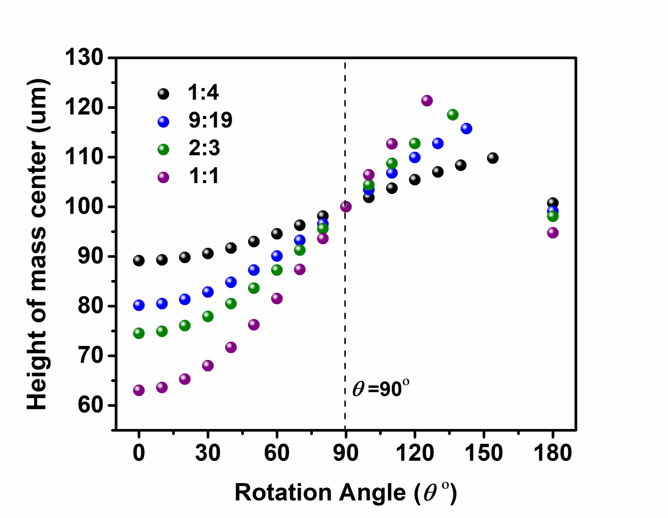

Figure S4. The height of mass center changes for various cavity sized particles at different rotation angle.

When the particle rotates an angle of θ around the Y_2 point, the center of mass G will raise and the corresponding potential energy will also increase as:

Potential energy change after rotation θ (Equation 3):

$$\Delta E_p(\theta) = \rho \cdot g \cdot V \cdot \Delta h = (\rho_{\mathrm{PEG}} - \rho_{\mathrm{water}}) \cdot g \cdot V \cdot (Y_2 - G) \cdot (1 - \cos\theta)$$

The density of PEGDA hydrogel is measured around 1.225 g/mL by Archimedes' immersion method.

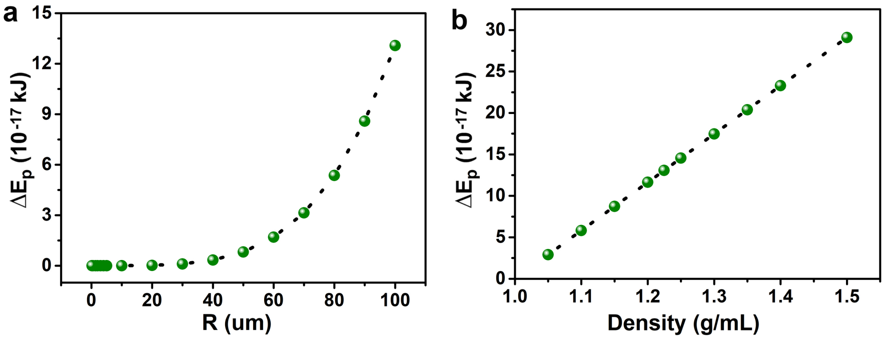

Figure S5. Potential energy change of particle (F_d: F_p=2: 3) with different (a) radius (*R*) and (b) density.

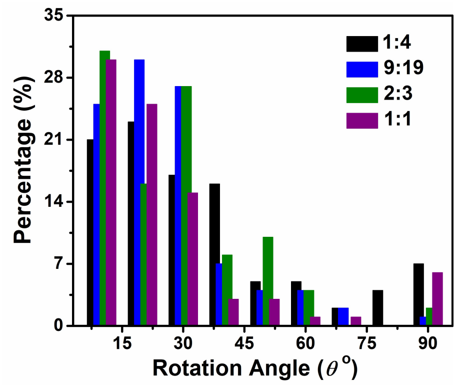

Figure S6. Rotation angle distributions of different cavity sized particles in aqueous solution after achieving equilibrium. The statistics are based on 400 crescent-shaped hydrogel microparticles (each kind of cavity sized particles are 100).

Friction. During the calculation, friction was approximately expressed according to the Stokes' law: $F_s=6\pi\eta av$ (where η is the viscosity of the solution, a and v are the radius and velocity of the particles). The rotation velocity is about 4°/s based on the time scale and frictional force is about $1.17*^{-11}$ N. The gravitational force is about $4.2*^{-8}$ N, which is much higher than frictional force. Thus, the gravity is the dominant factor of the spontaneous rotation.

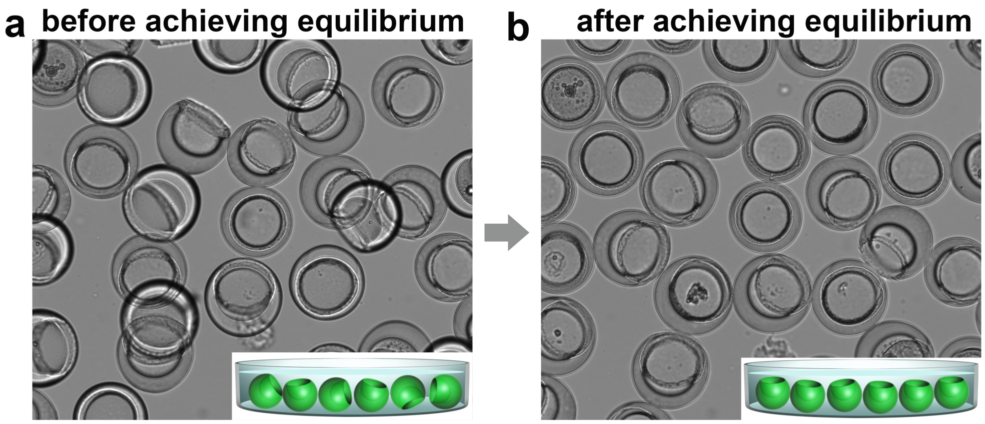

Figure S7. The orientation of crescent-shaped hydrogel microparticles before (a) and after (b) achieving equilibrium.

5. Partition coefficient of RGD peptide in ATPS

RGD peptide is cell-adhesive moieties which can be modified to PEG and dextran derivatives forming biomaterials for cell adhesion, spreading and proliferation. For the partition coefficient of RGD in ATPS, Dextran (28.6% W/W) and RGD peptide (2 mg/mL) were dissolved in 250 μL D_2O. The solution was mixed with 250 μL PEGDA D_2O solution (28.6% W/W). After phase separation, 150 μL PEGDA (up) and 150 μL dextran (down) were separately pipetted and diluted in two NMR tubes with 200 μL D_2O. 10 μL DMF was used as reference. Peptide in PEGDA phase and dextran phase were determined by ^{1}H NMR. As shown in **Figure S8**, the RGD peptide in PEGDA phase was much more than that in dextran phase and the partition coefficient of RGD peptide in ATPS (K_p) was calculated about 2.4. It indicates that the RGD peptide prefers PEGDA phase than dextran phase. Therefore, during the cross-linking reaction, RGD peptide can diffuse from dextran phase to PEGDA phase and be immobilized in the interior of the PEGDA hydrogel particles through the thiol-ene click reaction.

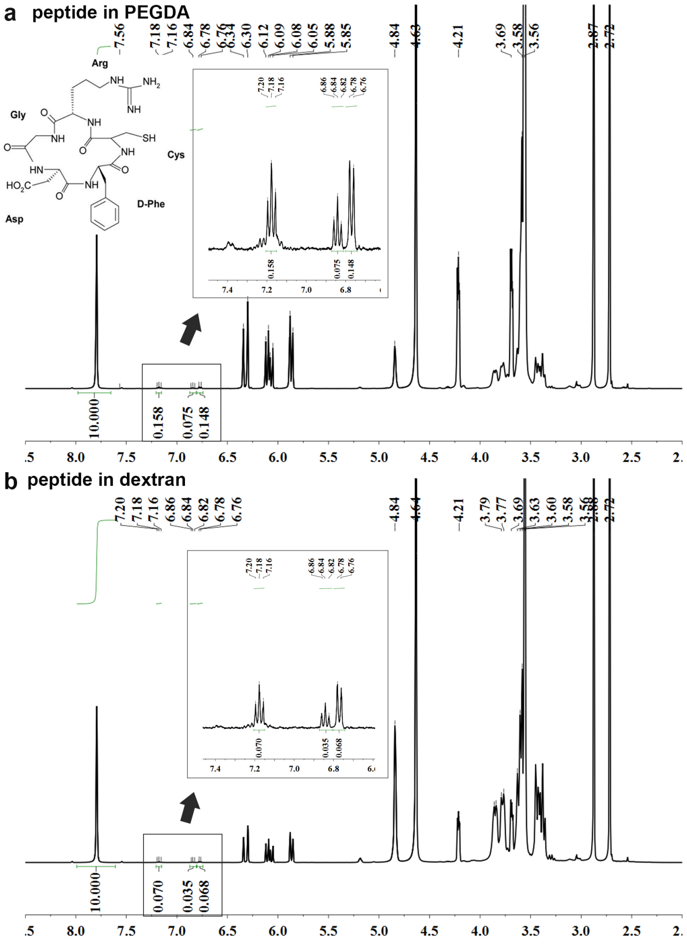

Figure S8. ^{1}H NMR of RGD peptide in ATPS of PEGDA phase (a) and dextran phase (b).

6. Bio experiment

Dulbecco's modified eagle medium (DMEM), newborn calf serum (NCS), penicillin/streptomycin (Pen-Strep) and Dulbecco's Phosphate Buffered Saline (DPBS) were purchased from Sigma-Aldrich (Steinheim, Germany). NIH/3T3 cells (mouse fibroblast cells) were obtained from American Type Culture Collection.

Cell staining. NIH/3T3 Cells were colored by CellTracker™ red CMTPX (Molecular Probes, C34552). Cells were detached from the T-flasks by 2 mL 0.25% Trypsin/2.21 mM EDTA and placed into 8 mL growth medium, containing 5 mL DMEM supplemented with 10% (v/v) NCS and 0.5% (v/v) Pen-Strep. 10 μL CellTracker™ Red CMTPX DMSO solution was also added into the growth medium, and cells are incubated at 37°C in 5%/95% CO_2/Air atmosphere. After 45 minutes, growth medium was removed by centrifugation.

Cell Culture. Cells were cultured in 25 cm^2 tissue culture flasks (T-flasks) and immersed in 5 mL growth medium. The crescent-shaped hydrogel microparticles were sterilized with 70% ethanol and washed with DPBS (3 times) and growth medium (3 times), respectively. Then particles with growth medium were added into sterile 8-well cell culture plate. Cells were trypsinized, stained and centrifuged to remove the growth medium. Cells were resuspended in new growth medium (around $5*10^4$ cells/mL) and added in the 8-well cell culture plate containing a layer of crescent-shaped hydrogel particles (as shown in **Figure S9**). The 8-well cell culture plate was placed in incubator.

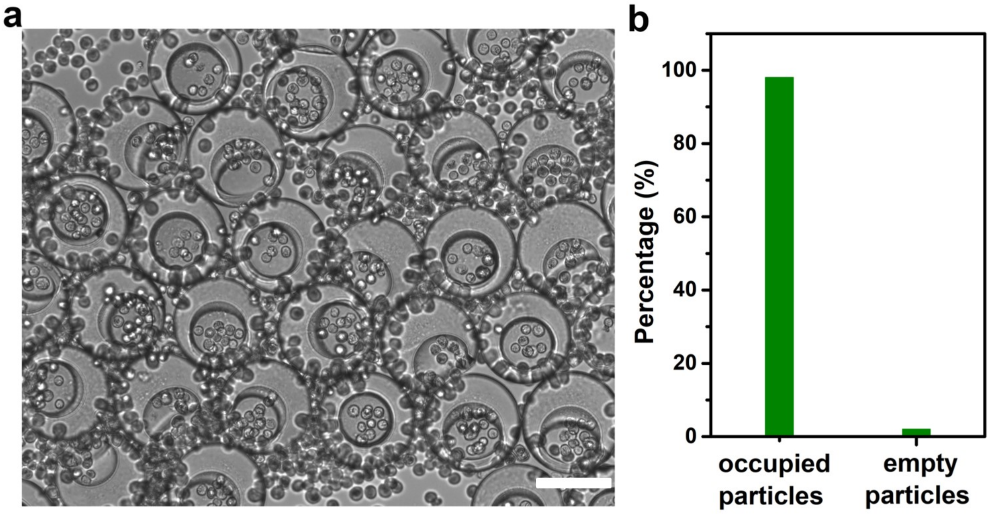

Figure S9. Cell loading (a) and occupied rate of 300 hydrogel particles (b). Scale bar 100 μm.

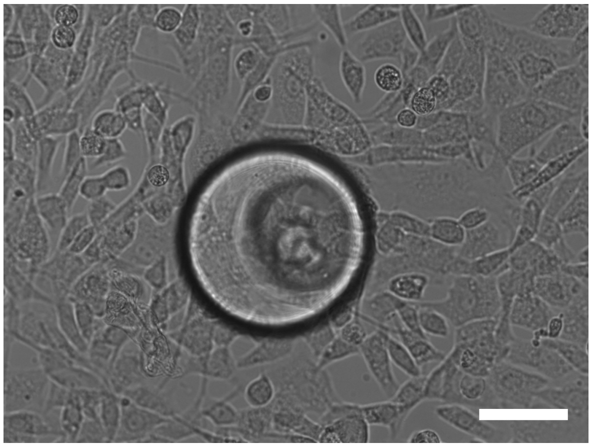

Figure S10. Cells outside the buckets. The cells adhere to and spread on the glass surface of the culture plate. Scale bar 50 μm.

Cell viability. Cell viability in the crescent-shaped hydrogel particles was checked through the double stain apoptosis detection kit. Hoechst 33342 and propidium iodide (PI) were used in the assay. Growth medium was removed from the culture plate which has hydrogel particles and cells. 0.004 mM Hoechst DPBS solution was added into the culture plate. The culture plate was incubated 10 min. Then, Hoechst solution was removed, and 75 mM PI DPBS solution was added into the culture plate. After 5 min, the sample was placed under Andor

inverted microscope and images from both channels were merged to observe the cells viability in hydrogel particles. Hoechst 33342 shows blue fluorescence (excitation/ emission maxima ~350/461 nm) when bound to DNA of cells. PI only permeates to dead cells with red fluorescence ((excitation/emission maxima ~535/617 nm). As shown in **Figure S11**, blue fluorescence was observed in all the cells, but no obvious red fluorescence and dead cells were observed.

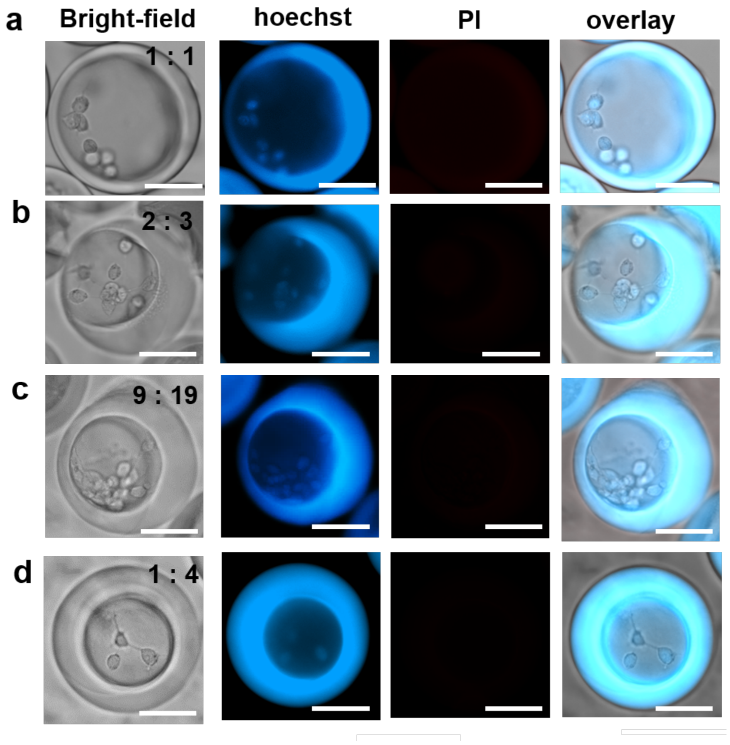

Figure S11. Cells viability in different cavity sized hydrogel particles. Scale bar 50 µm.

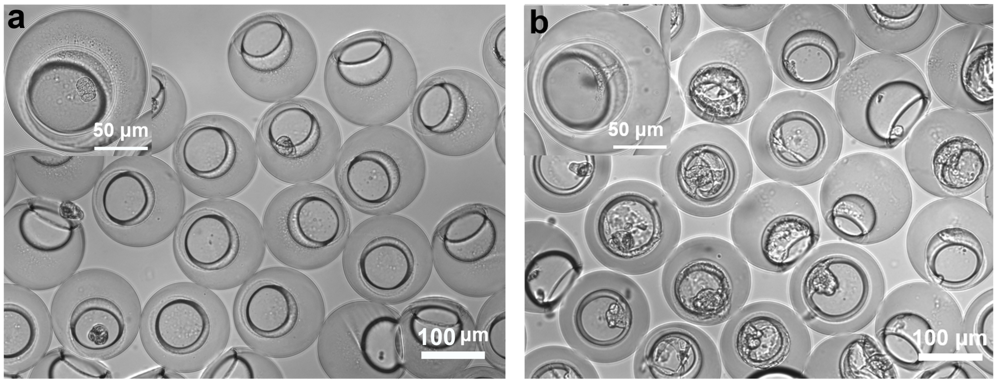

Figure S12. Cells in the unmodified particles (a) and peptide-modified particles (b) after transport. Most unmodified particles lost their cells after transport; however, most peptide-modified particles retained cells after transport.

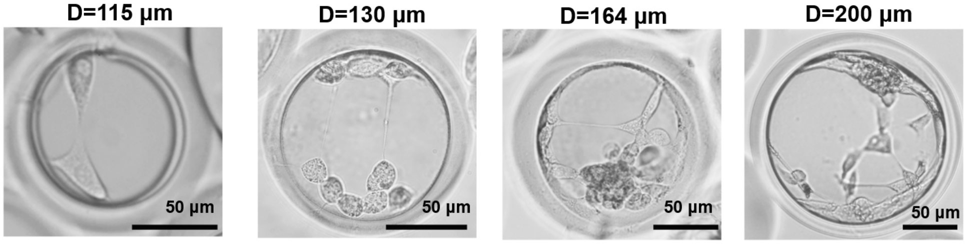

Figure S13. Cells in different sized hydrogel microparticle. The diameter is 115 µm, 130 µm, 164 µm and 200 µm (from left to right), respectively. Scale bar 50 µm. Cells adhered to the hydrogel, spread in the cavities and had cellular interactions in the crescent-shaped hydrogel microparticles.

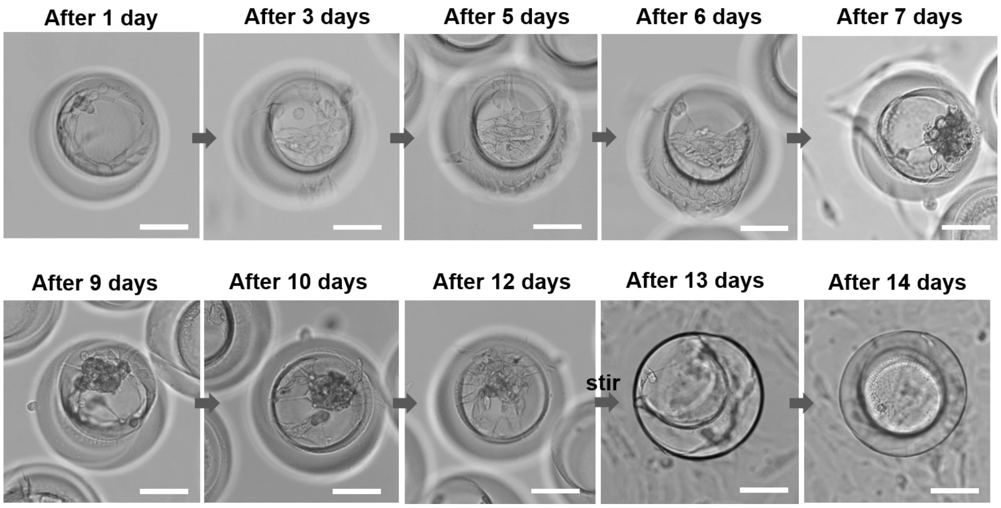

Figure S14. Cell proliferation and release in the hydrogel microparticle. Scale bar 50 μm. Cell proliferation was observed with time increase. After 3 days, cells migrated from cavity to outside. After 7 days, a few cells show up on the glass slide of culture plate, most cells still stayed in the hydrogel microparticle. After 13 days, cells were completely released from the microparticles by gentle agitation of the particle-cell suspensions for around 20 to 30 min. After cell release, the crescent hydrogel microparticle reoriented to the cavity facing up.

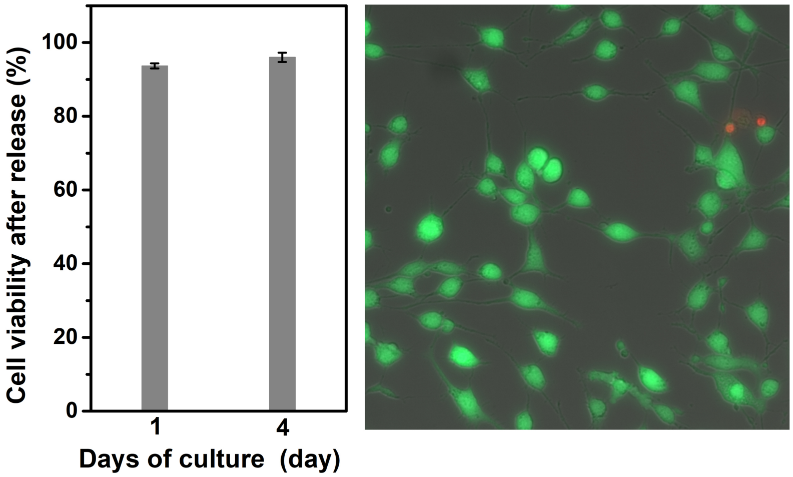

Figure S15. Cell viability of the cells after being released from the gel buckets. The cells were cultured from 1 day to 4 days after release, the cell viability was around 93%. Calcein AM (green) and propidium iodide (PI, red) were used to check cell live and death.

[1] T. Majima, W. Schnabel, W. Weber, *Macromol. Chem. Phys.* **1991**, 192, 2307-2315.
